# Factors that predict for representation of women in physician graduate medical education

**DOI:** 10.1080/10872981.2019.1624132

**Published:** 2019-06-14

**Authors:** Christina H. Chapman, Wei-Ting Hwang, Xingmei Wang, Curtiland Deville

**Affiliations:** a Department of Radiation Oncology, University of Michigan, Ann Arbor, MI, USA; b Center for Clinical Management Research, VA Ann Arbor Healthcare System, Ann Arbor, MI, USA; c Department of Biostatistics, Epidemiology and Informatics, University of Pennsylvania, Philadelphia, PA, USA; d Center for Clinical Epidemiology and Biostatistics, University of Pennsylvania, Philadelphia, PA, USA; e Department of Radiation Oncology and Molecular Radiation Sciences, Johns Hopkins, Baltimore, MD, USA

**Keywords:** Diversity, gender, graduate medical education, specialty selection, National Residency Match Program

## Abstract

**Background/Objective**: To identify factors associated with underrepresentation of women in the largest medical specialties.

**Methods**: The authors obtained specialty-specific data from the Association of American Medical Colleges, National Residency Match Program and Journal of the American Medical Association Graduate Medical Education Supplement from 2014 on the gender of trainees and faculty members, residency program director (PD)-rated importance of interview selection and rank list formation criteria, and characteristics of matched NRMP participants. They used linear regression to evaluate whether factors were associated with representation of female trainees in the 18 largest specialties that participated in the NRMP. They hypothesized that factors representing lower student exposure or higher research requirements would be associated with lower representation of women.

**Results**: In 2014, representation of women as trainees ranged from 13.7% in Orthopedic Surgery to 82.5% in OB/Gyn. On multivariable analysis, the factors associated with specialties having lower percentages of female trainees were: not being part of the third year core (slope = 0.141, p = 0.002), having lower specialty mean step 1 scores (slope = 0.007, p = 0.017), and having lower percentages of female faculty members. For each 1% increase in female faculty, the percentage of female trainees increased by 1.45% (p < 0.001).

**Conclusions**: Two exposure-related factors, percentage of female faculty members and being part of the third year core, were associated with underrepresentation of women as trainees. Future research could help examine whether these are causal associations. Medical schools and training specialties should investigate whether strategies to enhance mentorship and increase exposure to non-core specialties will increase the proportion of women in fields in which they are underrepresented.

## Introduction

Women are underrepresented as physicians in many specialties [1]. Because emerging evidence shows that the quality of female physician-led patient care is as good, and sometimes better [2,3], than that of male physicians, the underrepresentation of women suggests that specialties are missing out on talent. However, studies examining representation of women in medicine primarily focus on individual specialties and trends in representation without identifying causes.

Multiple factors may contribute to specialty-specific underrepresentation of women. Some may relate to broader societal issues (gender discrimination [4], work–life balance [5–7], and specific ways in which women would like to make a difference, including the importance of long-term patient relationships [5,8,9]). Others may involve the medical school and residency application process. Medical school and residency factors are important areas for intervention because they can likely be changed and standardized more easily than broader societal factors. They involve 1) exposure to [10] or 2) competitiveness of a specialty, with competitiveness being subcategorized as a) academic (grades and scores [11,12]) or b) research-related (publications, presentations and research masters/doctoral degrees) [13]. Factors that appear to be gender neutral may disproportionately affect women due to the influence of mentorship or other factors.

To investigate this issue further, we obtained data from the Association of American Medical Colleges (AAMC), American Medical Association (AMA), and National Residency Match Program (NRMP), on the gender of residents/fellows and medical school faculty, specialty-level data on characteristics of matched residency applicants, and specialty-specific program director-reported rankings of factors that influence residency applicant interview and rank process. We sought to investigate the relationships between representation of women in graduate medical education and a number of medical school and residency application factors that we organized into categories of academic competitiveness, research-related competitiveness and specialty exposure.

## Methods

*Data Sources and Measures*. GME trainee data were obtained from the 2013–2014 JAMA Graduate Medical Education Supplement [14]. We included the 20 largest specialties. We obtained data on female faculty representation from 2013 data from the AAMC FAMOUS Database [15], medical school factors from the 2014 National Resident Match Programs (NRMP) Charting Outcomes (CO) in the Match document [16], and residency program selection factors from the NRMP 2014 Program Directors’ (PD) Survey [17]. We excluded Ophthalmology and Urology due to lack of participation in the NRMP. Although we included Internal Medicine/Pediatrics residency in the analysis, there was no designation for dual board certification in the faculty data.

### Factor selection

**Figure 1.**
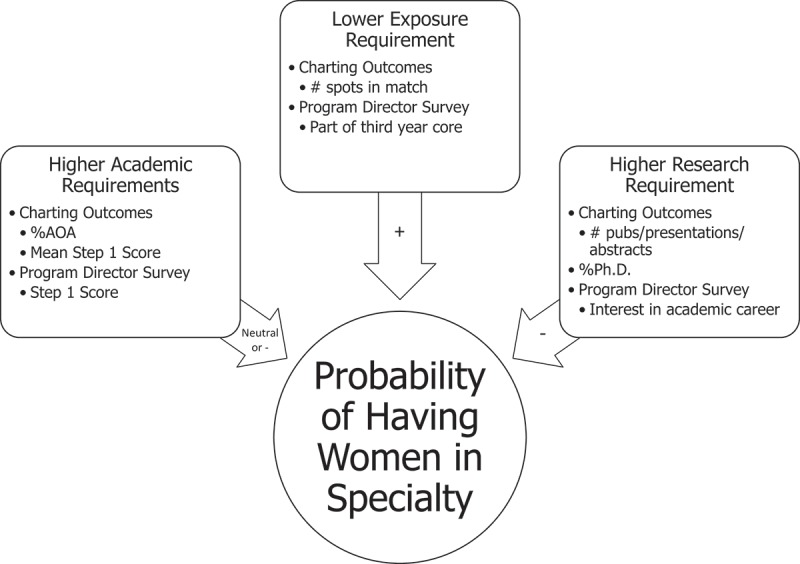
Conceptual model: conceptual model demonstrating the hypothesized relationships between the percentage of female trainees and factors related to exposure, grades, and research. Factors were chosen from either 2014 Charting Outcomes in the Match or the 2014 Residency Program Directors’ Survey.

We selected factors based on prior research and our de novo hypotheses about what might influence specialty gender representation, as shown in our conceptual model (Figure 1). Academic factors were: 1) percent of matched applicants in Alpha Omega Alpha honor society (%AOA), 2) mean step 1 score of matched applicants, and 3) PD-rated importance of step 1 score. Research-related factors were: 1) mean number of publications, presentations and abstracts of matched applicants, 2) percent of matched applicants with a PhD, and 3) PD-reported importance of demonstrated interest in an academic career. Exposure-related factors were: 1) CO total number of spots in the match (used as a surrogate for specialty size), 2) PD-rated audition rotation importance, and 3) being part of the third year core, a dichotomous variable defined as 1 if the specialty was any of the following: Internal Medicine, Obstetrics and Gynecology, Family Medicine, Psychiatry, Pediatrics, Surgery and Neurology [18].

### Statistical analysis

Individual-level data were not available. We therefore treated percentage of women per specialty as a continuous outcome variable, given that no specialty was comprised of <10% or >90% women. Medical school and residency factors were treated as continuous independent variables, except for part of the third year core, which was treated as a binary. We evaluated associations using linear regression models. We used the weighted least squares method to estimate the regression (e.g., slope) and correlation coefficients in order to take into account non-constant variance of the dependent variable (i.e., percentage of women) across different specialties, which is inversely proportional to the size of the specialty, ranging from 186 for Radiation Oncology to 6859 for Internal Medicine. We performed univariate regression first and included independent variables that had a p-value of < 0.1 in the multivariable regression. To explore whether a single specialty was driving certain association with female representation, we performed a sensitivity analysis excluding the specialties with the highest and lower percentages of women among those that had mean board scores in the top quartile: Dermatology (high percentage of women) and Orthopedic Surgery (low percentage of women). Statistical analyses were performed using Stata version 14. P-value<0.05 is considered statistically significant.

This study falls under the *** Institutional Review Board classification of ‘not regulated’ due to the use of publicly available data sources and lack of individual-level data.

## Results

Table 1 shows that characteristics of each specialty. The specialties with the lowest percentages of women were Orthopedic Surgery, Neurosurgery, Diagnostic Radiology, Radiation Oncology, and Otolaryngology.

**Table 1.** Characteristics of specialties that participate in National Residency Match Program, selected from 2014 Charting Outcomes in the March or the 2014 residency program directors’ survey.

Specialty	% Women 2013–2014 GME	# Women 2013–2014 JAMA GME	% Women Faculty AAMC 2013	# Women Faculty AAMC 2013	CO %AOA (matched)	CO Mean Step 1 score (matched US Grads)	Interview Step 1 Weighted Score	CO # Total Spots (unfilled spots included) 2014 Charting Outcomes	Part of Third Year Core	Interview Audition election/rotation within your department weighted score	CO Mean #pubs, abstracts, presentations (matched)	CO % with Ph.D (matched)	Interview Interest in academic career weighted score	Interview Applicant Was flagged with match violation by NRMP
Anesthesia	36.0%	2040	34.7%	2694	10.6%	230.0	4.455	1662	No	2.622	3.3	3.5%	0.864	2.205
Dermatology	64.0%	758	47.9%	631	51.0%	247.0	3.956	414	No	2.816	9.5	5.1%	2.009	0.833
Emergency Medicine	37.4%	2107	34.8%	1606	12.0%	230.0	3.783	1786	No	3.828	2.9	1.1%	1.05	1.334
Family Medicine	55.2%	5558	47.0%	2561	8.0%	218.0	3.458	3109	Yes	2.8	2.3	1.0%	0.256	1.824
IM/Peds	59.4%	856			22.0%	233.0	3.854	374	Yes	1.216	3.2	2.6%	0.54	1.152
Internal Medicine	43.5%	9984	33.0%	6570	16.0%	231.0	3.772	6859	Yes	1.944	3.9	4.2%	0.735	2.156
Neurology	44.7%	986	36.2%	1889	13.0%	230.0	3.6	723	Yes	2.36	4.9	8.9%	1.287	1.598
Neurosurgery	15.9%	202	17.4%	264	28.0%	244.0	4.092	206	No	2.814	11.7	12.8%	2.419	1.012
OB/GYN	82.5%	4079	56.9%	3164	13.0%	226.0	4.074	1242	Yes	2.418	3.3	1.7%	0.77	1.584
Orthopedic Surgery	13.7%	483	15.4%	507	32.0%	245.0	4.18	695	No	2.944	6.7	1.4%	0.95	0.96
Otolaryngology	34.3%	499	30.0%	600	39.0%	248.0	4.214	295	No	2.709	6.1	1.9%	1.47	1.152
Pathology	54.1%	1231	39.3%	2338	11.0%	231.0	4.042	597	No	2.496	5.6	22.0%	1.47	2.16
Pediatrics	73.1%	6233	53.3%	10,664	13.0%	226.0	3.92	2715	Yes	2.535	3.0	3.5%	0.648	1.44
PM&R	38.4%	446	45.8%	632	6.0%	220.0	3.64	391	No	2.881	3.3	2.5%	0.609	1.794
Psychiatry	54.2%	2666	46.7%	4820	5.0%	220.0	3.219	1374	Yes	2.665	3.8	4.5%	1.02	2.592
Radiation Oncology	28.7%	197	27.0%	409	24.0%	241.0	4.085	186	No	3.096	12.2	23.0%	2.12	1.081
Radiology-Diagnostic	27.2%	1218	27.3%	215	22.0%	241.0	4.158	1176	No	2.301	4.8	4.6%	0.891	1.932
Surgery-general	37.5%	2962	19.7%	298	15.0%	232.0	4.268	1210	Yes	2.12	4.4	2.0%	1.254	1.296
Total		42,505		39,862				24,640						

**Figure 2.**
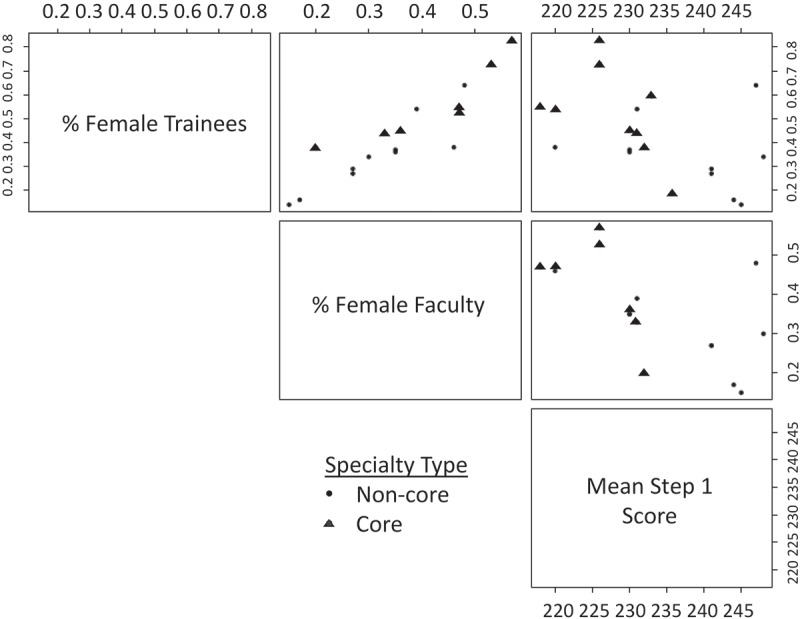
Scatter plot demonstrating univariate relationships between the percentage of female trainees, percentage of female faculty, and specialty mean step 1 score: Core specialties shown in blue and non-core specialties shown in black.

On univariate analysis, there was a positive correlation between female trainees and female faculty (slope 1.402, p < 0.001) and inclusion in the third year core (slope 0.225, p = 0.01). There was a negative correlation for mean step 1 score of matched US graduates (slope −0.012, p = 0.028, Figure 2). There was no association between the percentage of female trainees and: %AOA, importance of step 1 score for interview selection, number of match spots, importance of away rotation, importance of interest in an academic career, and % with a PhD (Table 2).

**Table 2.** Univariate and multivariable linear weighted least squares regression analysis with percentage of female trainees as the independent variable.

Variable	Weighted Least Squares		
2013–2014 GME	Slope	p-value	(95% CI)
**Univariate regression**			
2013% women faculty	1.402	<0.001	1.070,1.733
CO %AOA (matched)	−0.005	0.309	−0.014,0.005
CO Mean Step 1 score (matched US Grads)	−0.012	0.028	−0.022,−0.002
Interview Step 1 Weighted Score	−0.12	0.422	−0.429,0.189
CO # Total Spots (unfilled spots included)/100	0	0.838	−0.004,0.005
Part of Third Year Core	0.225	0.01	0.062,0.388
Interview Audition election/rotation	−0.07	0.452	−0.263,0.123
CO Mean #pubs, abstracts, presentations (matched)	−0.035	0.086	−0.076,0.006
CO % with Ph.D. (matched)	−0.005	0.607	−0.024,0.014
Interview Interest in academic career weighted	−0.14	0.15	−0.336,0.056
**Only variables with ***p*** ≤ 0.1 in the univariate regression**			
Variable			
2013% women faculty	1.451	<0.001	1.126,1.776
CO Mean Step 1 score (matched US Grads)	0.007	0.043	0.0003,0.014
Part of Third Year Core	0.140	0.003	0.058,0.221
CO Mean #pubs, abstracts, presentations (matched)	−0.003	0.780	−0.025,0.019
**Model with nonsignificant variables removed**			
2013% women faculty	1.450	<0.001	1.139,1.760
CO Mean Step 1 score (matched US Grads)	0.007	0.017	0.001,0.012
Part of Third Year Core	0.141	0.002	0.063,0.218

Multivariable regression revealed that for each 1% increase in women faculty, the percentage of female trainees increased by 1.45% (95% CI: 1.14–1.76, p < 0.001). Inclusion in the third year core remained significantly associated (slope 0.141, 95% CI: 0.06–0.22, p = 0.002) (Table 2). The slope for mean step 1 score changed from negative to positive, indicating that for each one-point increase in step 1 score, the percentage of female trainees increased by 0.007% (slope 0.007, 95% CI: 0.001, 0.012, p = 0.017). Mean Step 1 score and PD-rated step 1 score importance were highly collinear (correlation of 0.63), so the latter was dropped from the model. Mean characteristics of specialties based on whether they were or were not part of the third year core were as follows: % female trainees: 52.9% v. 33.6%, % female faculty: 44.1% v. 32.9%, mean numbers of publications, presentations, and abstracts: 3.5 v. 5.1, % with PhD, 3.3% v. 4.6%, step 1 score: 227 v 236 and % AOA: 22% v 25% (Table 3). Excluding Dermatology or Orthopedic surgery did not change the significance of trends described above.

**Table 3.** Aggregated characteristics of specialties based on whether they are or are not part of the third year core.

	Total # Matched US Seniors	% Women Trainees 2013–2014 GME	% Women Faculty AAMC 2013	CO %AOA (matched)	CO Mean Step 1 score (matched US Grads)	Mean# publications, presentations, and abstracts (matched applicants)	% with Ph.D. (matched applicants)
Not core	5300	33.6%	32.9%	25%	236	5.1	4.6%
Core	9637	52.9%	44.1%	22%	227	3.5	3.3%

## Discussion

Our study revealed that female trainee representation is positively associated with three factors: the specialty having a higher percentage of female faculty, higher mean board scores and being part of the third year core. Many of the factors that we hypothesized would be associated with gender representation were not, including metrics related to research productivity and away rotations. These trends were not driven by a single specialty. Our study design cannot confirm that each of these factors has causal relationships, but our findings provide support for future qualitative and quantitative work that may lead to the development of interventions to address specialty-specific underrepresentation of women.

### Female faculty representation

The correlation between female faculty and female trainee representation could simply reflect the fact that the underrepresentation of women in specific fields has persisted over time. However, previous research supports the notion that female trainees place importance on the presence of female residents [19], faculty [19,20] and role models [21], suggesting that female faculty underrepresentation may dissuade women from entering fields. This has been shown in Interventional Radiology [22]. The magnitude of the impact of gender diversity of specialty or program selection is less clear. Some studies show positive correlations (faculty [23], residents [24,25]), and others, no effect (chair [24,25], program director [25]).

Although increasing access to female mentorship is important, the importance of gender-concordance decreases when female mentees are able to develop personal connections with their mentors [26]. Therefore, emphasis should be also placed on understanding which features of gender-concordant mentoring relationships can be applied by male mentors. This can improve experiences for female trainees and ensure that mentoring responsibilities do not exclusively fall on women [27].

### Mandatory third year core rotation

Being part of the third year core predicts whether specialties are less than 40% women, with the exceptions being General Surgery (part of the core and 37.5% women) and Dermatology and Pathology (not part of the core and 64% and 54% women, respectively). Although underrepresentation of female faculty could contribute to female underrepresentation in non-core specialties, the association remained significant on multivariable analysis when accounting for female faculty representation. This suggests that women are affected by the lack of exposure to non-core fields in ways that men are not, even when accounting for the presence (or absence) of female faculty in these fields. The fact that specialty size was not correlated to trainee gender diversity suggests that it is not simply about how many physicians of a given specialty are present in society, but more closely related to whether students have mandatory exposure to those physicians.

Non-core fields were associated with higher applicant research productivity (Table 3), which is intrinsically tied to mentorship. Prior studies have consistently revealed gender-based discrimination in the pursuit of mentorship generally [28] and trainee research positions specifically [29]. Furthermore, female faculty have smaller publication networks, which affects academic rank [30]. The underlying causes of these publication network disparities might also affect female trainees at earlier points in their careers. If residency programs plan to continue to place strong emphasis on the number of publications and presentations, they should have an investment in mentorship at earlier stages, including pipeline programs [31–33] that involving structured shadowing and research opportunities.

Many medical schools are compressing their basic science curricula, allowing earlier clinical exposure and increased elective time. Schools should consider our findings and ensure broader specialty exposure for all students. Some curricula allow students to differentiate early based on their interests. These pathways may be ideal for students who have had already had adequate exposure to different areas, but could exacerbate gender disparities if women are informally expected to choose fields earlier on without exposure to small specialties and the few female role models that might exist within them. Additionally, non-core specialties appear to place greater emphasis on away rotations, which could allow for increased introduction of gender bias.

### Mean USMLE step 1 score

The third and final correlation noted between female representation in graduate medical education and mean step 1 scores was an interesting one. On univariate analysis, there was a negative association between representation of women and mean step 1 score, but this trend reversed on multivariable analysis, indicating that higher mean step 1 score was associated with increased representation of female trainees when controlling for the effects of female faculty representation and being part of the third year core. Though statistically significant, the magnitude of this finding was very small, and the overall significance is unclear. Male medical students have been shown to have slightly higher mean step 1 scores than women [12], but this finding does not hold for step 2, where women outperform men [11]. Understanding how mean step 1 scores of a specialty influence gender diversity is complex, because one must consider not only differences in the distribution of scores between men and women, but also whether 1) one gender is disproportionately deterred from applying to specialties having high mean step 1 scores and 2) program directors place equal emphasis on scores for both men and women. Further research that explores these issues is needed, as data are mixed regarding the utility of USMLE Step 1 and 2 scores in predicting success in training and practice [34–37]. Future studies should examine whether scores are predictive of residency performance once candidates surpass certain thresholds.

A number of factors that we hypothesized would be associated with specialty-specific gender representation were not, including metrics related to away rotations and research productivity. It is important to note that our study design does not exclude the possibility that these factors contribute to gender representation, either in the way that they affect medical students’ specialty selection choices or residency program directors interview and ranking process. The data collected by the NRMP may not sufficiently capture the nuances of student and program director decision-making. For example, differences in research productivity may contribute to gender inequity, but they could be driven by publication in high impact journals or in numbers of papers specifically, as opposed to the composite measure of the mean number of publications, presentations, and abstracts that is measured in Charting Outcomes. Survey and qualitative work may help yield better insight into the ways in which different selection criteria are used by students and residency programs. Strengths of our study include the use of national data on the largest medical specialties, increasing generalizability. Limitations of our study include the lack of individual-level data, which was not released due to confidentiality concerns, as well as the omission of fields that do not participate in the National Residency Match Program. Additionally, we did not use medical school level data, and as such we were unable to capture curricular variability across schools, given that some schools do mandate early exposure to certain fields that we designated as non-core.

## Conclusion

Not being part of the third year core, having lower percentages of female faculty, and having lower mean board scores are independently associated with specialties having lower percentages of female trainees. Future research should focus on determining whether these factors are causal and opposed to simply correlated. If these factors do indeed contribute to the underrepresentation of women, enhancing specialty-specific exposure and mentorship may be viable improvement strategies. These strategies likely need not be gender exclusive but should designed with inclusion of both female trainees and faculty in mind.

## Previous presentations

This manuscript was presented in part at the Association of American Medical Colleges 14^th^ Annual Health Workforce Research Conference on 12 May 2018.
